# Photocatalytic Hydrogen Production from Aqueous Solutions of Glucose and Xylose over Layered Perovskite-like Oxides HCa_2_Nb_3_O_10_, H_2_La_2_Ti_3_O_10_ and Their Inorganic-Organic Derivatives

**DOI:** 10.3390/nano12152717

**Published:** 2022-08-07

**Authors:** Sergei A. Kurnosenko, Vladimir V. Voytovich, Oleg I. Silyukov, Ivan A. Rodionov, Irina A. Zvereva

**Affiliations:** Department of Chemical Thermodynamics and Kinetics, Institute of Chemistry, Saint Petersburg State University, 199034 Saint Petersburg, Russia

**Keywords:** heterogeneous photocatalysis, hydrogen production, glucose, xylose, layered perovskite, titanate, niobate, intercalation, grafting

## Abstract

Nowadays, the efficient conversion of plant biomass components (alcohols, carbohydrates, etc.) into more energy-intensive fuels, such as hydrogen, is one of the urgent scientific and technological problems. The present study is the first one focused on the photoinduced hydrogen evolution from aqueous D-glucose and D-xylose using layered perovskite-like oxides HCa_2_Nb_3_O_10_, H_2_La_2_Ti_3_O_10_, and their organically modified derivatives that have previously proven themselves as highly active photocatalysts. The photocatalytic performance was investigated for the bare compounds and products of their surface modification with a 1 mass. % Pt cocatalyst. The photocatalytic experiments followed an innovative scheme including dark stages as well as the control of the reaction suspension’s pH and composition. The study has revealed that the inorganic−organic derivatives of the layered perovskite-like oxides can provide efficient conversion of carbohydrates into hydrogen fuel, being up to 8.3 times more active than the unmodified materials and reaching apparent quantum efficiency of 8.8%. Based on new and previously obtained data, it was shown that the oxides’ interlayer space functions as an additional reaction zone in the photocatalytic hydrogen production and the contribution of this zone to the overall activity is dependent on the steric characteristics of the sacrificial agent used.

## 1. Introduction

Plant biomass is one of the oldest and widely used sources of renewable energy. The latter is accumulated during photosynthesis, when plants convert the solar radiation into the energy of chemical bonds in organic compounds. Thus, targeted cultivation of plants for biomass can be considered a form of solar energy storage. Nevertheless, direct combustion of the plant biomass or its processing products does not appear to be sufficiently expedient because of their relatively low calorific value [1] and the release of carbon dioxide. With that said, there is particular interest in the biomass reforming to obtain more energy-intensive and environmentally friendly fuels such as hydrogen [2,3].

Currently, heterogeneous photocatalysis is being actively explored as a promising energy-saving method for hydrogen production from various sorts of organic raw [4]. In particular, the widely used sacrificial agents are primary alcohols (first of all, methanol) and amino alcohols (triethanolamine) [5,6]. However, these substances are not the only products of plant biomass processing. The other compounds to be reformed are carbohydrates, but significantly less attention is paid to their conversion into hydrogen fuel.

Nowadays, typical heterogeneous photocatalysts for carbohydrates reforming are composite materials on the basis of wide-gap semiconductor oxides (predominantly, TiO_2_ [7,8,9,10,11] as well as perovskites LaFeO_3_ [12,13]), sulfides (Zn_1−x_Cd_x_S [14], CdS/MoS_2_ [15,16]), tungstates (BiWO_6_ [17]), and metal-free compounds (g-C_3_N_4_ [18,19,20]). They were tested in relation to light-driven hydrogen production from glucose [21], xylose [22], fructose [23], sucrose [24], cellulose [25,26], and lignocellulose [27]. Further optimization of the hydrogen production conditions [28] as well as the creation of hybrid reactors, combining the hydrolysis of polysaccharides and their photocatalytic reforming [29], allowed making significant progress in this field. However, the photocatalytic activity, sufficient for the wide industrial use, has not been achieved yet, which justifies the development of new, more efficient materials.

A promising class of heterogeneous photocatalysts for hydrogen production is represented by ion-exchangeable layered perovskite-like oxides. Their crystal structure is formed by negatively charged perovskite layers of BO_6_ octahedra, alternating regularly with interlayer spaces occupied by cations. Ion-exchangeable layered perovskites are classified into two structural types: the Dion−Jacobson phases A′[A_n−1_B_n_O_3n+1_] and the Ruddlesden-Popper ones A′_2_[A_n−1_B_n_O_3n+1_], where A′ is the interlayer alkali cation, A is the alkaline earth or transition cation, and B is Ti, Nb, etc.) [30,31,32]. Outstanding photocatalytic properties of these oxides and their protonated forms (A′ = H) appear to be due to the unique structure of the perovskite octahedron BO_6_, providing efficient separation of photogenerated charge carriers, and active involvement of the interlayer space in intercalation and ion exchange reactions [33,34,35,36,37,38,39,40,41,42], including probable penetration of reactant molecules into this zone during photocatalytic processes [43,44]. The main approaches to the further improvement of layered perovskites’ photocatalytic performance are known to be partial ionic substitution (doping) in the perovskite octahedra [45,46,47,48,49], preparation of composites with solid cocatalysts and photosensitizers [50,51,52,53,54,55,56,57], creation of Z-schemes [58,59,60,61,62], sensitization with organic dyes [63], as well as exfoliation into separate nanolayers [64].

One of the key features of protonated layered perovskite-like oxides is their pronounced reactivity with respect to some organic compounds. This allows forming hybrid inorganic−organic derivatives, consisting of the layered inorganic matrix and organic modifiers, chemically anchored to the interlayer space via oxygen vertices of the perovskite octahedra [65,66,67]. Two key approaches to the synthesis of such hybrid materials are intercalation of organic bases [68,69,70] and esterification-like grafting of alcohols [71,72,73], carbohydrates [74], alkoxysilanes [75], and organophosphorus acids [76]. Despite the wide range of inorganic−organic derivatives synthesized, until recently, any data on their photocatalytic activity towards hydrogen production have been practically absent due to probable concerns about their stability under operating conditions [77]. However, our recent studies [78,79,80,81] have shown that oxides HCa_2_Nb_3_O_10_ and H_2_Ln_2_Ti_3_O_10_ (Ln = La, Nd), preliminarily modified by interlayer *n*-alkylamines and *n*-alkoxy groups, demonstrate outstanding photocatalytic performance with regard to light-driven hydrogen evolution from aqueous methanol in the near-ultraviolet range. Particularly, these inorganic−organic derivatives were found to be up to 117 times more active than the unmodified oxides and provide apparent quantum efficiency of more than 40% after additional surface platinization. Despite the fact that the interlayer organic modifiers experienced partial or, in some cases, even complete degradation upon photocatalysis, the activity of the samples was maintained throughout the whole measurement time, and hydrogen was proven to evolve from the reaction solution, not from the sample material. With that said, the photocatalytic behavior of organically modified layered perovskites appears to be an interesting and promising research direction. Particularly, of special interest is the applicability of these materials to hydrogen production from other biomass components, such as carbohydrates.

This paper presents the results of the investigation of layered perovskites HCa_2_Nb_3_O_10_, H_2_La_2_Ti_3_O_10_, and their organically modified derivatives as photocatalysts for the conversion of aqueous D-glucose and D-xylose into hydrogen for the first time. The choice of particular inorganic−organic derivatives for photocatalytic tests was based on the results of our previous studies [78,79,80,81]: for each oxide, we selected one *n*-alkylamine and one *n*-alkoxy derivative that demonstrated the highest efficiency of hydrogen evolution from aqueous methanol with a Pt cocatalyst (*n*-butylamine and ethoxy derivatives of HCa_2_Nb_3_O_10_, ethylamine and ethoxy ones of H_2_La_2_Ti_3_O_10_). This study also pays special attention to the influence of Pt reduction conditions on the photocatalytic activity as well as to the role of the oxides’ interlayer space in the hydrogen generation reactions.

## 2. Materials and Methods

### 2.1. Synthesis of Initial Protonated Oxides

Alkaline layered perovskite-like oxides KCa_2_Nb_3_O_10_ (KCN_3_) and K_2_La_2_Ti_3_O_10_ (KLT_3_) were synthesized in accordance with the conventional ceramic method using preliminarily calcined Nb_2_O_5_, CaO, TiO_2_, La_2_O_3_, and K_2_CO_3_ as reactants (Vekton, Saint Petersburg, Russia). The oxides were weighed in stoichiometric amounts, and potassium carbonate was taken with a 30% excess to compensate for the loss during calcination. To prepare the reaction mixture, the reactants were placed into a grinding bowl with silicon nitride balls and ground under an *n*-heptane layer in a Fritsch Pulverisette 7 planetary micro mill (Fritsch, Idar-Oberstein, Germany) at a rotation speed of 600 rpm, using a program of 10 repetitions of 10 min each separated by 5 min intervals. The mixture obtained was dried and pelletized into ~2 g tablets at a pressure of 50 bar using an Omec PI 88.00 hydraulic press (Omec, Certaldo, Italy). Then, the tablets were placed into corundum crucibles with lids, calcined in a Nabertherm L-011K2RN muffle furnace (Nabertherm GmbH, Lilienthal, Germany) and, after cooling down, ground in an agate mortar. The temperature program of KCN_3_ synthesis consisted of two stages (800 °C for 12 h and 1100 °C for 24 h) with intermediate grinding and re-pelletizing. KLT_3_ was prepared via one-stage heating (1100 °C for 12 h).

To obtain the protonated niobate HCa_2_Nb_3_O_10_∙yH_2_O (HCN_3_), the KCN_3_ powder was treated with 12 M nitric acid at a ratio of 100 mL per 5 g of the sample at 25 °C for 1 day. After this, the product was centrifuged, thoroughly rinsed with water to remove acid residues and dried under ambient pressure. To avoid dehydration, HCN_3_ was further stored in an atmosphere of humid air. To prepare the protonated titanate H_2_La_2_Ti_3_O_10_ (HLT_3_), the KLT_3_ powder was kept in the atmosphere of humid air for 1 day. The hydrated alkaline titanate obtained was then treated with water at a ratio of 200 mL per 1 g of the sample for 1 h, centrifuged and stirred in 0.1 M hydrochloric acid, taken at the same ratio, for 1 day. Then, the final product was separated via centrifugation in a laboratory centrifuge ELMI CM-6MT (ELMI, Riga, Latvia) and dried in a desiccator over CaO for 2 days.

### 2.2. Synthesis of Inorganic−Organic Derivatives

The organic modification of the protonated oxides was performed in accordance with the methods developed and optimized in our previous reports [79,80,81].

To prepare the *n*-butylamine derivative HCN_3_×BuNH_2_ of the niobate, 0.25 g of HCN_3_, 9 mL of *n*-butylamine (Chemical line, Saint Petersburg, Russia), and 1 mL of distilled water were mixed in a sealed tube and stirred at 25 °C for 1 day. The ethoxy derivative HCN_3_×EtOH was synthesized under solvothermal conditions. For this, 1 g of HCN_3_ was mixed with 35 mL of a 96% azeotropic aqueous ethanol solution in a sealed PTFE vessel that than was inserted into a steel laboratory autoclave and, after intense shaking, kept at 100 °C for 7 days.

Ethylamine HLT_3_×EtNH_2_ and ethoxy HLT_3_×EtOH derivatives of the titanate were synthesized according to the strategy of successive replacement of some organic modifiers by others, since direct synthesis of the required samples in a pure single-phase state did not appear possible. HLT_3_×EtNH_2_ was prepared using the methylamine derivative HLT_3_×MeNH_2_ as a precursor, which, in turn, was obtained from the protonated titanate. For this, 1 g of the protonated sample was placed into a glass tube with 30 mL of 38% aqueous methylamine (Chemical line, Saint Petersburg, Russia), whereupon the mixture was sonicated on an ultrasonic homogenizer Hielscher UP200St (Hielscher, Teltow, Germany) at a half power for 5 min. Then, the tube was sealed, and the suspension was stirred at 60 °C for 10 days. The methylamine precursor was filtered, mixed with 30 mL of 70% aqueous ethylamine (Merck, Darmstadt, Germany) and stirred 25 °C for 1 day to obtain the target ethylamine derivative HLT_3_×EtNH_2_. The ethoxy derivative HLT_3_×EtOH was prepared on the basis of the *n*-butylamine precursor HLT_3_×BuNH_2_, which, in turn, was synthesized via a 1 day stirring of 1 g of the methylamine one in a 90% aqueous *n*-butylamine solution at 25 °C. One gram of the *n*-butylamine sample was mixed with 35 mL of a 96% azeotropic aqueous ethanol solution in a sealed polytetrafluoroethylene (PTFE) vessel that then was inserted into a steel laboratory autoclave and, after intense shaking, kept at 180 °C for 7 days.

All the final products were filtered and rinsed with acetone to remove residual organic reactants adsorbed on the surface. The synthesis conditions described are summarized in Table 1.

### 2.3. Investigation of Photocatalytic Activity

Photocatalytic activity was studied with respect to light-driven hydrogen production from 1 mol. % solutions of D-glucose and D-xylose in water for both bare samples and products of their modification with a 1% Pt cocatalyst under near-ultraviolet irradiation. The measurements were performed on the laboratory photocatalytic setting used in our previous reports [78,79,80,81] and described in detailes in Supporting Information Figure S1. The hydrogen formation rate ω, apparent quantum efficiency *ϕ*, and multiplicity of increase in the rate after Pt loading (platinization increase factor k_Pt_) were chosen as quantitative indicators of the photocatalytic performance. The method for calculation *ϕ* is presented in Supporting Information Figure S2. Before the photocatalytic tests, the direct photolysis of the aqueous carbohydrates was investigated via their irradiation through a light filter in the absence of a catalyst and the detection of hydrogen evolved.

#### 2.3.1. Testing the Activity of Bare Samples (No Cocatalyst)

To prepare the reaction suspension, 0.03 g of the sample were placed in a round-bottom flask containing 60 mL of 1 mol. % aqueous D-glucose or D-xylose. The flask was sealed, shaken and sonicated in an Elmasonic S10H bath (Elma, Singen, Germany) for 10 min. After this, 54 mL of the suspension obtained were pumped into the reaction compartment of the cell followed by turning on a magnetic stirrer, a light filter, a lamp, and an argon flow through the suspension. After 15 min, 4 mL of the suspension was taken from the cell to establish an actual volume concentration of the sample and pH of the medium before the photocatalytic measurement (c_1_, pH_1_). After 15 min, an argon purging of the reaction compartment was turned off, and the photocatalytic measurement, consisting in chromatographic analysis of the gaseous phase every 15 min, was conducted for 2 h. Afterwards, the lamp was turned off to organize a 45 min dark stage and monitor the potential activity of the sample in the absence of irradiation. Thereafter, 4 mL of the suspension were sampled to determine the volume concentration of the sample and pH of the medium at the end of the photocatalytic measurement (c_2_, pH_2_). After this, 30 mL of the residual suspension were centrifuged at a separation factor F = 1000 for 1 h to precipitate bulk particles and analyze the liquid phase composition (pH_3_).

#### 2.3.2. Testing the Activity of Pt-Loaded Samples

Two ways of surface platinization were investigated: in situ Pt reduction in a 1 mol. % aqueous carbohydrate and preliminary reduction in 1 mol. % aqueous methanol. In the first case, 53 mL of the initial suspension in a carbohydrate were pumped into the reaction compartment. Fifteen minutes after turning on the stirrer, the light filter, the lamp, and argon, 1.1 mL of the 2.56 mM H_2_PtCl_6_ aqueous solution were injected into the reaction suspension to perform the photocatalytic platinization of the sample’s surface. The solution volume was calculated to provide the mass fraction of Pt in the photocatalyst of 1% assuming its full reduction. In the second case, the sample was initially dispersed in 53 mL of 1 mol. % aqueous methanol, platinized as described above, separated via filtering and then redispersed in a 1 mol. % carbohydrate.

The products of the direct in situ platinization were further designated as “sample/Pt” and those of the platinization in methanol as “sample/Pt(MeOH)”. In the study of all the platinized samples, the gas phase was analyzed every 5 min, and the dark stage duration was 20 min. Other experimental conditions and procedures were the same.

### 2.4. Instrumentation

#### 2.4.1. XRD

Powder X-ray diffraction (XRD) analysis of the samples was performed on a Rigaku Miniflex II benchtop diffractometer (Rigaku, Tokyo, Japan) using CuK_α_ radiation, an angle range of 2*θ* = 3°–60°, a scanning rate of 10°/min, and a step of 0.02°. The lattice parameters in the tetragonal system were calculated on the basis of all the diffraction peaks observed using the DiffracPlus Topas 4.2 software (Bruker, Karlsruhe, Germany).

#### 2.4.2. Spectrophotometry

Spectrophotometric analyses of the photocatalytic suspensions were performed on a Thermo Scientific Genesys 10S UV–Vis spectrophotometer (Thermo Fisher Scientific, Waltham, MA, USA). Measurements were conducted in the range of optical density A of <2 using 1 mol. % aqueous D-glucose or D-xylose for dilution and baseline recording. Actual volume concentrations of the samples (c_1_, c_2_) were calculated by means of previously built calibration plots (Supporting Information Figure S3).

#### 2.4.3. pH Measurement

The pH values of a reaction suspensions’ medium were determined using a laboratory pH-meter Toledo SevenCompact S220 (Mettler-Toledo GmbH, Greifensee, Switzerland) equipped with an InLab Expert Pro-ISM electrode.

#### 2.4.4. Other Methods of Analysis

The comprehensive characterization of the samples in question by means of Raman spectroscopy, ^13^C nuclear magnetic resonance (^13^C NMR), diffuse reflectance spectroscopy (DRS), thermogravimetry (TG), elemental CHN analysis, scanning electron microscopy (SEM), and Brunauer–Emmett–Teller method (BET) is presented in our previous reports [78,79,80,81].

## 3. Results and Discussion

### 3.1. Characterization of the Protonated Oxides and Their Inorganic−Organic Derivatives

**Figure 1:**
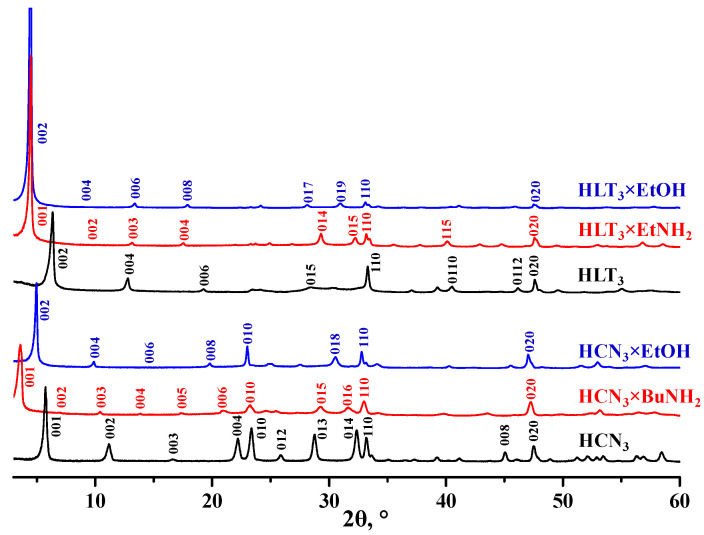
XRD patterns of the samples obtained via the protonation of initial oxides (HLT_3_ and HCN_3_), subsequent intercalation of amines (HCN_3_×RNH_2_ and HLT_3_×RNH_2_), and grafting of alcohols (HCN_3_×ROH and HLT_3_×ROH).

The initial protonated oxides HCN_3_ and HLT_3_ as well as their alkylamine and alkoxy derivatives were identified by means of powder XRD analysis (Figure 1). The indexing of the patterns has revealed the successful synthesis of all the target compounds in a pure form without perceptible by-phases. Although the organic modification proceeded as a topochemical reaction (occurring with the preservation of the layered perovskite structure in general), the derivatives obtained were new individual crystalline phases, which caused the differences in the XRD patterns as compared to the initial materials. The main transformations observed from the XRD data were the expansion of the interlayer space by the organic components being inserted and change in the conformation (relative arrangement) of adjacent perovskite slabs during the formation of some derivatives. The aforementioned expansion of the layered structure along the *c* crystallographic axis is seen from the low-angle shift of the (00x) reflections and the corresponding increase in the *c* lattice parameter and interlayer distance *d* (Table 2), which is measured between the centers of adjacent perovskite layers. The perovskite conformation change may be predicted based on the different relation between *c* and *d* values in the initial oxide and its derivative. For instance, HCN_3_, HCN_3_×BuNH_2_, and HLT_3_×EtNH_2_ appeared to exist in an eclipsed conformation (without a relative shift of adjacent perovskite slabs, *c* = *d*), whilst HLT_3_, HLT_3_×EtOH, and HCN_3_×EtOH were probably stacked in a staggered conformation (with a relative shift of the slabs along lateral axes by *a*/2, *c* = 2*d*). Thus, the relative arrangement of adjacent perovskite slabs, apparently, changed in the course of ethanol grafting into the niobate and ethylamine intercalation into the titanate. At the same time, positions of some reflections, such as (110), (010), and (020), and the *a* lattice parameter hardly experienced perceptible changes upon the organic modification pointing to the preservation of the perovskite layer structure.

As shown in our previous reports [78,79,80,81], the alkylamine derivatives synthesized represented inorganic−organic intercalates with alkylammonium cations, associated with interlayer oxygen anions, and the alkoxy ones were those with organic chains grafted covalently to the perovskite matrix. In addition to the organic modifier, all the derivatives also contained some amounts of interlayer water (Table 2). Despite the aforementioned interlayer expansion, the bandgap energy of the inorganic−organic samples hardly differed much from that of the initial protonated oxides, since energy bands of the materials in question are known to be formed by the atoms of perovskite octahedra [82]. Thus, the operation ranges of both protonated and organically modified oxides were near ultraviolet. Taking into account the DRT-125 lamp spectrum (Supporting Information Figure S4), the differences in the long-wavelength absorption edge of HLT_3_, HCN_3_, HCN_3_×BuNH_2_, and HCN_3_×EtOH samples (Table 2) cannot be a weighty reason for those in their photocatalytic performance, since the lamp did not have emission bands in the range of 343–360 nm. The red absorption edges of HLT_3_×EtNH_2_ (366 nm) and HLT_3_×EtOH (364 nm) were located near an intense lamp peak at 365 nm, which could cause a higher photocatalytic activity of these samples due to a greater amount of available light. However, since the 365 nm emission peak coincided with the optical absorption edge of the samples, the quantum yield of the photocatalytic reaction at this wavelength was scarcely high. With that said, the possible absorption at 365 nm was not taken into account, while calculating the quantum efficiency *ϕ* and, consequently, its value for inorganic−organic derivatives of HLT_3_ may be overestimated.

The specific surface areas of the samples (Table 2) were relatively low by the standards of heterogeneous photocatalysts, which is predominantly due to the ceramic synthesis of the alkaline precursors, providing a high crystallinity but strong intergrowth of the polycrystals. Nevertheless, the inorganic−organic derivatives in question have demonstrated impressive photocatalytic performance with respect to hydrogen production from aqueous alcohols, probably due to the unique layered structure and active involvement of the interlayer space in promoting the target reaction [78,79,80,81].

### 3.2. Photocatalytic Activity with Respect to Carbohydrates Reforming

**Figure 2:**
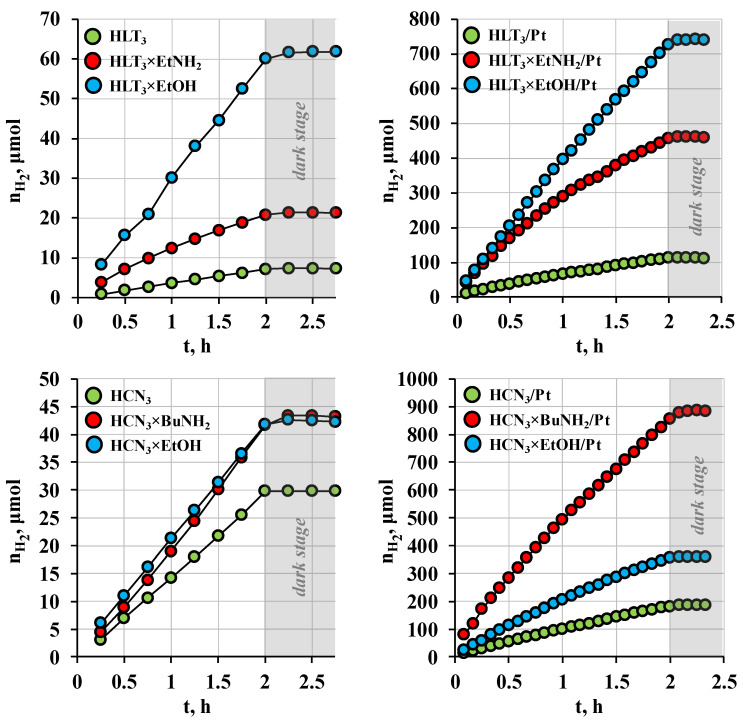
Kinetic curves of hydrogen generation from 1 mol. % aqueous D-glucose over the protonated oxides, inorganic−organic derivatives, and products of their in situ platinization under near ultraviolet irradiation.

The photocatalytic measurements were aimed at studying the kinetics of hydrogen evolution from 1 mol. % solutions of D-glucose and D-xylose in water, the influence of the platinization method on the catalytic performance, the stability of the reaction suspensions, and changes in their composition during photocatalysis. Dark stages organized at the end of each experiment (Figure 2 and Supporting Information Figures S5–S7) proved the photocatalytic nature of the hydrogen evolution reactions, since the reaction rate became zero as soon as the radiation source was switched off.

Preliminary experiments on the ultraviolet irradiation (*λ* > 220 nm) of the aqueous carbohydrates in the absence of a catalyst revealed that D-glucose and D-xylose underwent direct photolysis, accompanied by hydrogen formation at rates of 2.7 and 3.2 μmol/h, respectively (Supporting Information Figure S5). The use of bare protonated oxides HCN_3_ and HLT_3_ as photocatalysts allowed one to improve the hydrogen generation rate up to 5.1 times under the same irradiation conditions, achieving apparent quantum efficiencies of *ϕ* = 0.2% for HCN_3_ and *ϕ* = 0.1% for HLT_3_ (Table 3). As in methanol [80,81], the photocatalytic performance of the niobate in D-glucose turns out to be much greater than that of the titanate. A potential reason for this difference is high hydratability of the HCN_3_’s interlayer space (HCN_3_·1.5H_2_O), which is not typical of HLT_3_. The intercalated water may serve as a source of reactive hydroxyl radicals, involved in the oxidation of a sacrificial agent along with photogenerated holes and additionally increasing the photocatalytic reaction rate. The photocatalytic in situ platinization of the protonated niobate and titanate allowed increasing the rate of D-glucose conversion into hydrogen by approximately 7 and 17 times, respectively (Table 3). Photoreduced Pt nanoparticles function as a cocatalyst, facilitating surface charge separation and creating active sites for hydrogen evolution [83]. Meanwhile, the in situ Pt reduction was found not to be an optimal method for the platinization of the protonated oxides. The latter, previously platinized in aqueous methanol, exhibited a 1.5–1.8 times greater performance in the reaction of D-glucose reforming (Table 3), which pointed to a better reducing ability of relatively small methanol in comparison with that of the bulky carbohydrate.

All the inorganic−organic derivatives obtained showed an enhanced photocatalytic activity in both carbohydrates as compared with in the initial protonated oxides (Figure 2). The most active sample among bare ones tested in D-glucose was the ethoxy derivative HLT_3_×EtOH (*ϕ* = 0.50%) being superior to the protonated titanate HLT_3_ by 8.3 times. After in situ platinization, the greatest activity values were exhibited by the *n*-butylamine derivative of the niobate HCN_3_×BuNH_2_/Pt (*ϕ* = 7.5% in D-glucose and *ϕ* = 8.8% in D-xylose). Thus, the organically modified samples represent promising photocatalytic materials for hydrogen production, not only from methanol solutions but also from those of carbohydrates.

The rigorous evaluation of the photocatalytic activity achieved in relation to the results reported earlier was complicated by the fact that different research groups tested the samples’ performance under unequal conditions (light source, catalyst and carbohydrate concentrations, cocatalyst loading, etc.), which made it impossible to compare directly hydrogen evolution rates in the absence of quantum efficiency data. In general, the activity of organically modified and platinized samples towards hydrogen production from D-glucose and D-xylose (Table 3) exceeded that demonstrated by TiO_2_-based photocatalysts (TiO_2_/Pt and TiO_2_/RuO_2_/Pt) under similar experimental conditions or had a comparable value [7,21]. At the same time, it is important to keep in mind that widely used TiO_2_ P25 Degussa and its derivatives have an order of magnitude greater specific surface area than layered perovskite-like oxides presented in this study. As a consequence, the latter have the advantage of the activity normalized per unit area, even if the gross activity of a TiO_2_-based material proves to be higher.

The available experimental data still did not allow interpreting strictly the derivatives’ increased photocatalytic performance. However, taking into account structural features of these compounds and the hypothesis about their interlayer space as an additional reaction zone in photocatalysis, we can associate the aforementioned activity increase with the greater accessibility of the organically modified interlayer space for reactants. It has been shown on different photocatalytic materials [84,85,86] that the dissociative adsorption of alcohols yielding grafted alkoxy groups is an important step of their photooxidation. In our case, this process is likely to occur in the interlayer space of the modified oxide, which is supported by the very fact of the successful synthesis of grafted derivatives. Despite the partial or, in some cases, even complete decomposition of inserted organic components during photocatalysis [79,80,81], the interlayer reaction zone appeared to remain expanded and continue functioning at least as long as the sample was in the reaction medium, which accounted for the increased activity. Moreover, the enlarged interlayer distance *d* of the inorganic−organic derivatives could facilitate the penetration and reduction of H_2_PtCl_6_ within the interlayer zone forming there new active sites of hydrogen evolution.

Unlike the protonated oxides, all the inorganic−organic samples platinized in situ demonstrated greater activity than those decorated with Pt previously in aqueous methanol (Table 3). In view of the above, a reason for this difference may be the aforementioned degradation of interlayer organic modifiers during irradiation in aqueous methanol followed by the irreversible interlayer space contraction upon the sample separation and drying. In this case, the interlayer distance, apparently, was restored, which limited the supply of reactants into the interlayer zone and thereby decreased the activity.

No matter whether the interlayer organic modifiers decomposed or not, the hydrogen was formed from the carbohydrate solution, not from the sample itself, which was confirmed by the following calculations. For instance, the ethoxy derivative HLT_3_×EtOH/Pt tested in D-glucose gave 745 μmol H_2_ throughout the 2 h photocatalytic experiment, which exceeded 8.6 times the H_2_ amount that could have been formed potentially in the course of the complete decomposition of the grafted ethoxy groups, contained in 25 mg of the sample (86.5 μmol H_2_), while the activity was maintained. Thus, the reaction of hydrogen production indeed can be considered catalytic.

**Figure 3:**
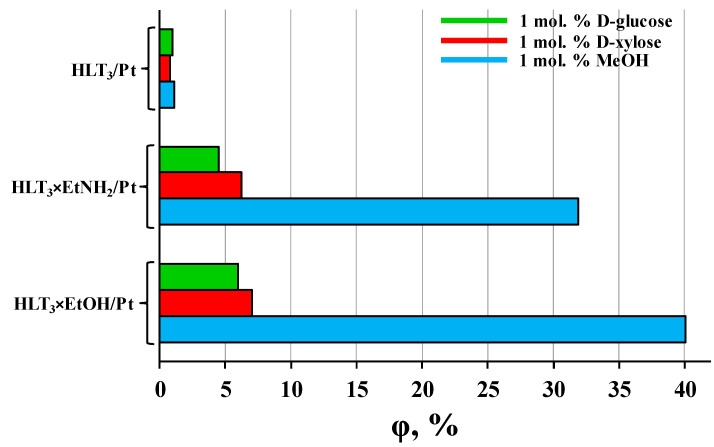
Comparison of the apparent quantum efficiency of hydrogen evolution from aqueous solutions of carbohydrates and methanol over platinized HLT_3_-based photocatalysts.

**Figure 4:**
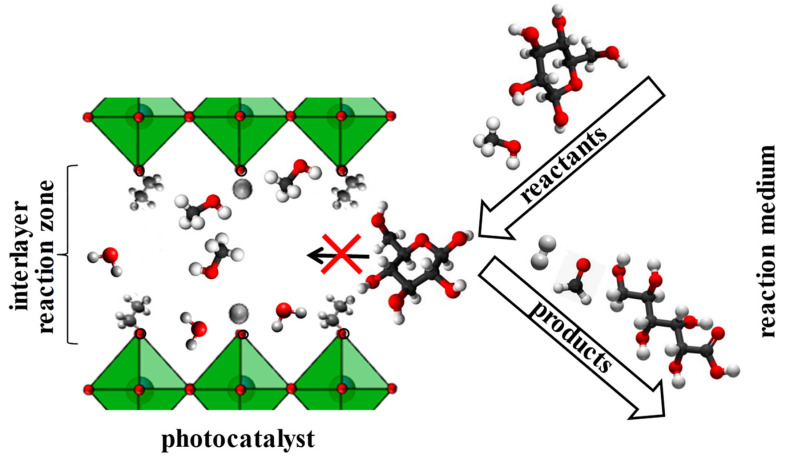
Interlayer space as an additional reaction zone in photocatalytic hydrogen generation.

Another evidence of the participation of the inorganic−organic derivatives’ interlayer space in photocatalytic reactions is a strong correlation of their activity with the reactant molecules’ size (Figure 3). For the example of the HLT_3_-based inorganic−organic samples, it was clearly seen that the quantum efficiency of hydrogen production increased by 5–7 times when going from aqueous carbohydrates to aqueous methanol. At the same time, the performance of the unmodified protonated titanate increased no more than 1.5 times and thereby depended on the specific sacrificial agent’s size rather weakly. These experimental facts indicated that the interlayer space indeed functions as an additional reaction zone but the contribution of this zone in total photocatalytic performance is expressly dependent on the steric characteristics of reactants (at close values of redox potentials). For instance, relatively small methanol molecules readily penetrate the interlayer space and undergo oxidation while bulky D-glucose ones react predominantly at the boundary of the interlayer space with the reaction medium and on the external crystal surface (Figure 4).

According to the data on the analysis of the photocatalytic suspensions (Supporting Information Figures S8 and S9), the inorganic−organic derivatives (especially alkylamine ones) demonstrated good dispersibility in aqueous carbohydrates and provided noticeably greater volume concentrations than the initial protonated oxides taken in the same amount. However, HCN_3_-based samples formed more stable suspensions than HLT_3_-based ones: the latter precipitated more intensely on the cell walls despite the continuous stirring, because of which the actual volume concentrations decrease during the photocatalytic measurement by two or more times. This can explain deviations of some kinetic curves from a linear course.

Most of the studied samples provided weakly acidic pH values of the reaction medium (Supporting Information Figure S8), which should have a beneficial effect on the hydrogen production rate. Unlike aqueous methanol [79,80,81], solutions of carbohydrates appeared to suppress the deintercalation of amines from the alkylamine derivatives, since the latter gave suspensions with subacidic or at least neutral pH.

**Figure 5:**
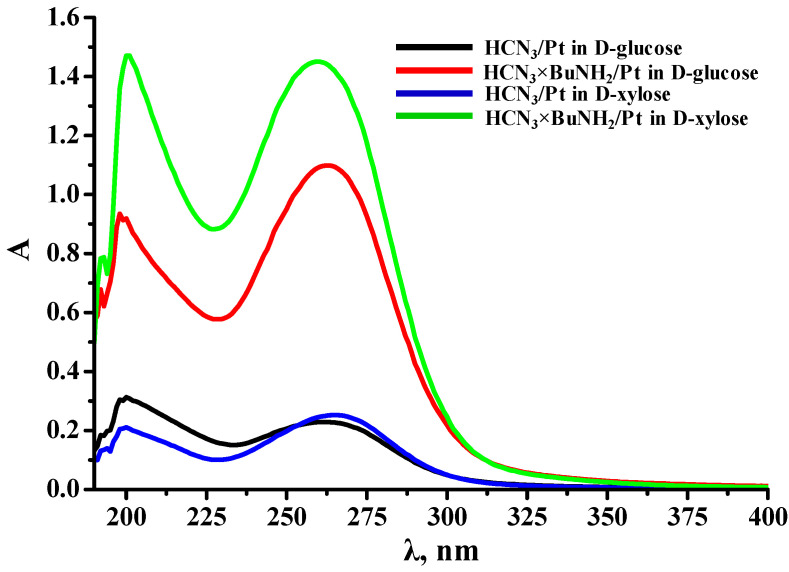
Ultraviolet absorption spectra of the reaction solutions after photocatalytic experiments (5× dilution; solid samples were separated via centrifugation).

Generally, pH values of the reaction medium decreased during photocatalytic hydrogen generation (Supporting Information Figure S8). In addition, the final reaction solutions analyzed after centrifugation (Figure 5) showed pronounced absorption bands in the near ultraviolet region, which were not associated with residual suspended particles since the solutions did not exhibit the Tyndall effect being illuminated by a laser. Moreover, the peak intensities generally correlated with the photocatalytic performance of the corresponding samples. The aforementioned pH reduction and appearance of the spectra indicated that the aldonic acids were one (but apparently not the only) of the liquid-phase products of the carbohydrates reforming [87].

## 4. Conclusions

The present study has shown that organically modified layered perovskite-like oxides HCN_3_ and HLT_3_ can be used for the efficient photocatalytic conversion of aqueous carbohydrates into hydrogen fuel. The derivatives were superior in the photocatalytic activity to the unmodified oxides up to 8.3 times and, after surface platinization, exhibited apparent quantum efficiency up to 8.8%. However, the in situ platinization of the organically modified samples provided greater catalytic performance than that preliminarily conducted in aqueous methanol. Experimental data indicated that the interlayer space of the organically modified photocatalysts functions as an additional reaction zone in the photocatalytic hydrogen production and the contribution of this zone to the overall activity is dependent on the steric characteristics of the sacrificial agent used.

## Figures and Tables

**Table 1:** Conditions for the synthesis of the inorganic−organic derivatives.

Sample	Precursor	Organic Content in the Reaction Mixture (vol. %)	Temperature (°C)	Duration (d)
HCN_3_×BuNH_2_	HCN_3_	90	25	1
HCN_3_×EtOH	HCN_3_	96	100	7
HLT_3_×EtNH_2_	HLT_3_×MeNH_2_	70	25	1
HLT_3_×EtOH	HLT_3_×BuNH_2_	96	180	7

**Table 2:** Lattice parameters in the tetragonal system, interlayer distances *d*, quantitative compositions (interlayer organic *x* and water *y* content per formula unit), light absorption edge, and specific surface areas of the samples.

Sample	*a* (Å)	*c* (Å)	*d* (Å)	*x*	*y*	*E_g_* (eV)	*λ*_max_ (nm)	*S* (m^2^/g)
HCN_3_	3.82	16.0	16.0	−	1.50	3.50	354	7.6
HCN_3_×BuNH_2_	3.86	25.4	25.4	1.00	0.45	3.62	343	5.0
HCN_3_×EtOH	3.86	35.8	17.9	0.90	0.45	3.50	354	3.9
HLT_3_	3.79	27.2	13.6	−	0.15	3.44	360	3.2
HLT_3_×EtNH_2_	3.82	20.2	20.2	0.70	0.35	3.39	366	− *
HLT_3_×EtOH	3.83	39.5	19.8	0.85	0.40	3.41	364	− *

* not measured.

**Table 3:** Photocatalytic activity of the protonated oxides and inorganic−organic derivatives.

	Sample	*ω* (μmol/h)	*ϕ* (%)	*k*_Pt_
**D-glucose**	HCN_3_	14	0.2	−
	HCN_3_/Pt	95	1.6	7
	HCN_3_/Pt(MeOH)	140	2.4	10
	HCN_3_×BuNH_2_	21	0.4	−
	HCN_3_×BuNH_2_/Pt	450	7.5	21
	HCN_3_×BuNH_2_/Pt(MeOH)	410	6.8	20
	HCN_3_×EtOH	20	0.3	−
	HCN_3_×EtOH/Pt	200	3.3	10
	HCN_3_×EtOH/Pt(MeOH)	170	2.8	9
	HLT_3_	4	0.1	−
	HLT_3_/Pt	61	1.0	15
	HLT_3_/Pt(MeOH)	110	1.8	28
	HLT_3_×EtNH_2_	10	0.2	−
	HLT_3_×EtNH_2_/Pt	270	4.5	27
	HLT_3_×EtNH_2_/Pt(MeOH)	240	4.0	24
	HLT_3_×EtOH	30	0.5	−
	HLT_3_×EtOH/Pt	360	6.0	12
	HLT_3_×EtOH/Pt(MeOH)	120	2.0	4
**D-xylose**	HCN_3_/Pt	88	1.5	−
	HCN_3_×BuNH_2_/Pt	530	8.8	−
	HCN_3_×EtOH/Pt	140	2.3	−
	HLT_3_/Pt	48	0.80	−
	HLT_3_×EtNH_2_/Pt	380	6.3	−
	HLT_3_×EtOH/Pt	420	7.0	−

## Data Availability

The data presented in this study are available in the article body and Supplementary Materials.

## Supplementary Materials

The following supporting information can be downloaded at https://www.mdpi.com/article/10.3390/nano12152717/s1, Figure S1: Scheme and operation principle of photocatalytic setting, Figure S2: Spectrophotometric calibrations for the express measurement of reaction suspensions’ concentrations, Figure S3: Emission spectrum of the DRT-125 mercury lamp measured through the employed light filter solution compared with the absorption regions of the photocatalysts under study, Figure S4: Kinetic curves of photolytic hydrogen evolution from 1 mol. % aqueous carbohydrates under ultraviolet irradiation, Figure S5: Kinetic curves of photocatalytic hydrogen generation from 1 mol. % aqueous D-glucose over HCN_3_- and HLT_3_-based samples previously platinized in aqueous methanol, Figure S6: Kinetic curves of photocatalytic hydrogen generation from 1 mol. % aqueous D-xylose over HCN_3_- and HLT_3_-based samples platinized in situ, Table S1: Actual volume concentrations of the samples in suspensions and pH of the reaction medium, Figure S7: Comparison of the actual concentrations of the samples in the reaction suspensions at the beginning and the end of the photocatalytic measurement.

## References

[1] Davis K.A., Yoo S., Shuler E.W., Sherman B.D., Lee S., Leem G. (2021). Photocatalytic hydrogen evolution from biomass conversion. Nano Converg..

[2] Omri M., Sauvage F., Golonu S., Wadouachi A., Pourceau G. (2018). Photocatalyzed transformation of free carbohydrates. Catalysts.

[3] Yao Y., Gao X., Li Z., Meng X. (2020). Photocatalytic reforming for hydrogen evolution: A review. Catalysts.

[4] Kennedy J., Bahruji H., Bowker M., Davies P.R., Bouleghlimat E., Issarapanacheewin S. (2018). Hydrogen generation by photocatalytic reforming of potential biofuels: Polyols, cyclic alcohols, and saccharides. J. Photochem. Photobiol. A Chem..

[5] Rodionov I.A., Zvereva I.A. (2016). Photocatalytic activity of layered perovskite-like oxides in practically valuable chemical reactions. Russ. Chem. Rev..

[6] Wang S., Zhang J., Li B., Sun H., Wang S. (2021). Engineered Graphitic Carbon Nitride-Based Photocatalysts for Visible-Light-Driven Water Splitting: A Review. Energy Fuels.

[7] Bellardita M., García-López E.I., Marcì G., Palmisano L. (2016). Photocatalytic formation of H_2_ and value-added chemicals in aqueous glucose (Pt)-TiO_2_ suspension. Int. J. Hydrog. Energy.

[8] Zhou M., Li Y., Peng S., Lu G., Li S. (2012). Effect of epimerization of D-glucose on photocatalytic hydrogen generation over Pt/TiO_2_. Catal. Commun..

[9] Bahadori E., Ramis G., Zanardo D., Menegazzo F., Signoretto M., Gazzoli D., Pietrogiacomi D., Michele A.D. (2020). Photoreforming of Glucose over CuO/TiO_2_. Catalysts.

[10] Bellardita M., García-López E.I., Marcì G., Nasillo G., Palmisano L. (2018). Photocatalytic Solar Light H_2_ Production by Aqueous Glucose Reforming. Eur. J. Inorg. Chem..

[11] Iervolino G., Vaiano V., Murcia J.J., Rizzo L., Ventre G., Pepe G., Campiglia P., Hidalgo M.C., Navío J.A., Sannino D. (2016). Photocatalytic hydrogen production from degradation of glucose over fluorinated and platinized TiO_2_ catalysts. J. Catal..

[12] Iervolino G., Vaiano V., Sannino D., Rizzo L., Ciambelli P. (2016). Production of hydrogen from glucose by LaFeO_3_ based photocatalytic process during water treatment. Int. J. Hydrog. Energy.

[13] Iervolino G., Vaiano V., Sannino D., Rizzo L., Galluzzi A., Polichetti M., Pepe G., Campiglia P. (2018). Hydrogen production from glucose degradation in water and wastewater treated by Ru-LaFeO_3_/Fe_2_O_3_ magnetic particles photocatalysis and heterogeneous photo-Fenton. Int. J. Hydrog. Energy.

[14] Zhao H., Li C.F., Yong X., Kumar P., Palma B., Hu Z.Y., van Tendeloo G., Siahrostami S., Larter S., Zheng D. (2021). Coproduction of hydrogen and lactic acid from glucose photocatalysis on band-engineered Zn_1-x_Cd_x_S homojunction. iScience.

[15] Li C., Wang H., Ming J., Liu M., Fang P. (2017). Hydrogen generation by photocatalytic reforming of glucose with heterostructured CdS/MoS_2_ composites under visible light irradiation. Int. J. Hydrog. Energy.

[16] Zheng X., Wang X., Liu J., Fu X., Yang Y., Han H., Fan Y., Zhang S., Meng S., Chen S. (2021). Construction of NiP_x_/MoS_2_/NiS/CdS composite to promote photocatalytic H_2_ production from glucose solution. J. Am. Ceram. Soc..

[17] Madriz L., Tatá J., Carvajal D., Núñez O., Scharifker B.R., Mostany J., Borrás C., Cabrerizo F.M., Vargas R. (2020). Photocatalysis and photoelectrochemical glucose oxidation on Bi_2_WO_6_: Conditions for the concomitant H_2_ production. Renew. Energy.

[18] Speltini A., Scalabrini A., Maraschi F., Sturini M., Pisanu A., Malavasi L., Profumo A. (2018). Improved photocatalytic H_2_ production assisted by aqueous glucose biomass by oxidized g-C_3_N_4_. Int. J. Hydrog. Energy.

[19] Bai X., Hou Q., Qian H., Nie Y., Xia T., Lai R., Yu G., Laiq Ur Rehman M., Xie H., Ju M. (2022). Selective oxidation of glucose to gluconic acid and glucaric acid with chlorin e6 modified carbon nitride as metal-free photocatalyst. Appl. Catal. B Environ..

[20] Speltini A., Romani L., Dondi D., Malavasi L., Profumo A. (2020). Carbon nitride-perovskite composites: Evaluation and optimization of photocatalytic hydrogen evolution in saccharides aqueous solution. Catalysts.

[21] Kawai T., Sakata T. (1980). Conversion of carbohydrate into hydrogen fuel by a photocatalytic process. Nature.

[22] Kurenkova A.Y., Markovskaya D.V., Gerasimov E.Y., Prosvirin I.P., Cherepanova S.V., Kozlova E.A. (2020). New insights into the mechanism of photocatalytic hydrogen evolution from aqueous solutions of saccharides over CdS-based photocatalysts under visible light. Int. J. Hydrog. Energy.

[23] Kondarides D.I., Patsoura A., Verykios X.E. (2010). Anaerobic photocatalytic oxidation of carbohydrates in aqueous Pt/TiO_2_ Suspensions with simultaneous production of hydrogen. J. Adv. Oxid. Technol..

[24] Beasley C., Gnanamani M.K., Qian D., Hopps S.D. (2021). Photocatalytic Reforming of Sucrose and Dextrose for Hydrogen Production on Pd/TiO_2_. ChemistrySelect.

[25] Caravaca A., Jones W., Hardacre C., Bowker M. (2016). H_2_ production by the photocatalytic reforming of cellulose and raw biomass using Ni, Pd, Pt and Au on titania. Proc. R. Soc. Math. Phys. Eng. Sci..

[26] Chang C., Skillen N., Nagarajan S., Ralphs K., Irvine J.T.S., Lawton L., Robertson P.K.J. (2019). Using cellulose polymorphs for enhanced hydrogen production from photocatalytic reforming. Sustain. Energy Fuels.

[27] Kuehnel M.F., Reisner E. (2018). Solar Hydrogen Generation from Lignocellulose. Angew. Chem. Int. Ed..

[28] Ramis G., Bahadori E., Rossetti I. (2020). Design of efficient photocatalytic processes for the production of hydrogen from biomass derived substrates. Int. J. Hydrog. Energy.

[29] Zou J., Zhang G., Xu X. (2018). One-pot photoreforming of cellulosic biomass waste to hydrogen by merging photocatalysis with acid hydrolysis. Appl. Catal. A Gen..

[30] Schaak R.E., Mallouk T.E. (2002). Perovskites by Design: A Toolbox of Solid-State Reactions. Chem. Mater..

[31] Uppuluri R., Sen Gupta A., Rosas A.S., Mallouk T.E. (2018). Soft chemistry of ion-exchangeable layered metal oxides. Chem. Soc. Rev..

[32] Tani S., Komori Y., Hayashi S., Sugahara Y. (2006). Local environments and dynamics of hydrogen atoms in protonated forms of ion-exchangeable layered perovskites estimated by solid-state ^1^H NMR. J. Solid State Chem..

[33] Silyukov O., Chislov M., Burovikhina A., Utkina T., Zvereva I. (2012). Thermogravimetry study of ion exchange and hydration in layered oxide materials. J. Therm. Anal. Calorim..

[34] Silyukov O.I., Kurnosenko S.A., Zvereva I.A. (2018). Intercalation of Methylamine into the Protonated Forms of Layered Perovskite-Like Oxides HLnTiO_4_ (Ln = La and Nd). Glas. Phys. Chem..

[35] Kurnosenko S.A., Silyukov O.I., Mazur A.S., Zvereva I.A. (2020). Synthesis and thermal stability of new inorganic-organic perovskite-like hybrids based on layered titanates HLnTiO_4_ (Ln = La, Nd). Ceram. Int..

[36] Shelyapina M.G., Lushpinskaya I.P., Kurnosenko S.A., Silyukov O.I., Zvereva I.A. (2020). Identification of Intercalates and Grafted Organic Derivatives of H_2_La_2_Ti_3_O_10_ by Multinuclear NMR. Russ. J. Gen. Chem..

[37] Kurnosenko S.A., Silyukov O.I., Minich I.A., Zvereva I.A. (2021). Exfoliation of Methylamine and *n*-Butylamine Derivatives of Layered Perovskite-Like Oxides HLnTiO_4_ and H_2_Ln_2_Ti_3_O_10_ (Ln = La, Nd) into Nanolayers. Glas. Phys. Chem..

[38] Silyukov O.I., Minich I.A., Zvereva I.A. (2018). Synthesis of Protonated Derivatives of Layered Perovskite-Like Bismuth Titanates. Glas. Phys. Chem..

[39] Minich I.A., Silyukov O.I., Gak V.V., Borisov E.V., Zvereva I.A. (2020). Synthesis of Organic–Inorganic Hybrids Based on Perovskite-like Bismuth Titanate H_2_K_0.5_Bi_2.5_Ti_4_O_13_·H_2_O and *n*-Alkylamines. ACS Omega.

[40] Shelyapina M.G., Silyukov O.I., Lushpinskaia I.P., Kurnosenko S.A., Mazur A.S., Shenderovich I.G., Zvereva I.A. (2020). NMR Study of Intercalates and Grafted Organic Derivatives of H_2_La_2_Ti_3_O_10_. Molecules.

[41] Silyukov O.I., Kurnosenko S.A., Minich I.A., Rodionov I.A., Zvereva I.A. (2021). Protonated Forms of Layered Perovskite-Like Titanate NaNdTiO_4_: Neutron and X-ray Diffraction Structural Analysis. Solids.

[42] Shelyapina M.G., Silyukov O.I., Andronova E.A., Nefedov D.Y., Antonenko A.O., Missyul A., Kurnosenko S.A., Zvereva I.A. (2021). ^1^H NMR Study of the HCa_2_Nb_3_O_10_ Photocatalyst with Different Hydration Levels. Molecules.

[43] Rodionov I.A., Silyukov O.I., Utkina T.D., Chislov M.V., Sokolova Y.P., Zvereva I.A. (2012). Photocatalytic properties and hydration of perovskite-type layered titanates A_2_Ln_2_Ti_3_O_10_ (A = Li, Na, K; Ln = La, Nd). Russ. J. Gen. Chem..

[44] Takata T., Furumi Y., Shinohara K., Tanaka A., Hara M., Kondo J.N., Domen K. (1997). Photocatalytic Decomposition of Water on Spontaneously Hydrated Layered Perovskites. Chem. Mater..

[45] Zou Z., Ye J., Arakawa H. (2001). Substitution effects of In^3+^ by Fe^3+^ on photocatalytic and structural properties of Bi_2_InNbO_7_ photocatalysts. J. Mol. Catal..

[46] Reddy V., Hwang D., Lee J. (2003). Effect of Zr substitution for Ti in KLaTiO_4_ for photocatalytic water splitting. Catal. Lett..

[47] Kumar V., Govind, Uma S. (2011). Investigation of cation (Sn^2+^) and anion (N^3-^) substitution in favor of visible light photocatalytic activity in the layered perovskite K_2_La_2_Ti_3_O_10_. J. Hazard. Mater..

[48] Zhou Y., Wen T., Guo Y., Yang B., Wang Y. (2016). Controllable doping of nitrogen and tetravalent niobium affords yellow and black calcium niobate nanosheets for enhanced photocatalytic hydrogen evolution. RSC Adv..

[49] Kawashima K., Hojamberdiev M., Chen S., Yubuta K., Wagata H., Domen K., Teshima K. (2017). Understanding the effect of partial N^3−^-to-O^2−^ substitution and H^+^-to-K^+^ exchange on photocatalytic water reduction activity of Ruddlesden–Popper layered perovskite KLaTiO_4_. Mol. Catal..

[50] Huang Y., Li J., Wei Y., Li Y., Lin J., Wu J. (2009). Fabrication and photocatalytic property of Pt-intercalated layered perovskite niobates H_1−x_LaNb_2−x_Mo_x_O_7_ (x = 0–0.15). J. Hazard. Mater..

[51] Huang Y., Li Y., Wei Y., Huang M., Wu J. (2011). Photocatalytic property of partially substituted Pt-intercalated layered perovskite, ASr_2_Ta_x_Nb_3−x_O_10_ (A = K, H; x = 0, 1, 1.5, 2 and 3). Sol. Energy Mater. Sol. Cells.

[52] Oshima T., Wang Y., Lu D., Yokoi T., Maeda K. (2019). Photocatalytic overall water splitting on Pt nanocluster-intercalated, restacked KCa_2_Nb_3_O_10_ nanosheets: The promotional effect of co-existing ions. Nanoscale Adv..

[53] Cui W., Qi Y., Liu L., Rana D., Hu J., Liang Y. (2012). Synthesis of PbS–K_2_La_2_Ti_3_O_10_ composite and its photocatalytic activity for hydrogen production. Prog. Nat. Sci. Mater. Int..

[54] Cui W., Liu L., Ma S., Liang Y., Zhang Z. (2013). CdS-sensitized K_2_La_2_Ti_3_O_10_ composite: A new photocatalyst for hydrogen evolution under visible light irradiation. Catal. Today.

[55] Cui W., Guo D., Liu L., Hu J., Rana D., Liang Y. (2014). Preparation of ZnIn_2_S_4_/K_2_La_2_Ti_3_O_10_ composites and their photocatalytic H_2_ evolution from aqueous Na_2_S/Na_2_SO_3_ under visible light irradiation. Catal. Commun..

[56] Saito K., Kozeni M., Sohmiya M., Komaguchi K., Ogawa M., Sugahara Y., Ide Y. (2016). Unprecedentedly enhanced solar photocatalytic activity of a layered titanate simply integrated with TiO_2_ nanoparticles. Phys. Chem. Chem. Phys..

[57] Liu Y., Zhou Y., Lv C., Zhang C., Jin X., Meng Q., Chen G. (2018). Construction of 2D-composite HCa_2_Nb_3_O_10_ /CaNb_2_O_6_ heterostructured photocatalysts with enhanced hydrogen production performance. New J. Chem..

[58] Chen X., Shen S., Guo L., Mao S.S. (2010). Semiconductor-based photocatalytic hydrogen generation. Chem. Rev..

[59] Zhang L., Wong K.H., Chen Z., Yu J.C., Zhao J., Hu C., Chan C.Y., Wong P.K. (2009). AgBr-Ag-Bi_2_WO_6_ nanojunction system: A novel and efficient photocatalyst with double visible-light active components. Appl. Catal. A Gen..

[60] Kim H.G., Jeong E.D., Borse P.H., Jeon S., Yong K., Lee J.S., Li W., Oh S.H. (2006). Photocatalytic Ohmic layered nanocomposite for efficient utilization of visible light photons. Appl. Phys. Lett..

[61] Kim H.G., Borse P.H., Choi W., Lee J.S. (2005). Photocatalytic Nanodiodes for Visible-Light Photocatalysis. Angew. Chem..

[62] Zhang L., Wang G., Xiong Z., Tang H., Jiang C. (2018). Fabrication of flower-like direct Z-scheme β-Bi_2_O_3_/g-C_3_N_4_ photocatalyst with enhanced visible light photoactivity for Rhodamine B degradation. Appl. Surf. Sci..

[63] Youngblood W.J., Anna Lee S.H., Maeda K., Mallouk T.E. (2009). Visible light water splitting using dye-sensitized oxide semiconductors. Acc. Chem. Res..

[64] Maeda K., Mallouk T.E. (2018). Two-Dimensional Metal Oxide Nanosheets as Building Blocks for Artificial Photosynthetic Assemblies. Bull. Chem. Soc. Jpn..

[65] Sanchez P.G.-R. (2006). and C. Functional Hybrid Materials.

[66] Kickelbick G. (2007). Hybrid Materials: Synthesis, Characterization, and Applications.

[67] Mir S.H., Nagahara L.A., Thundat T., Mokarian-Tabari P., Furukawa H., Khosla A. (2018). Review—Organic-Inorganic Hybrid Functional Materials: An Integrated Platform for Applied Technologies. J. Electrochem. Soc..

[68] Constantino V.R.L., Barbosa C.A.S., Bizeto M.A., Dias P.M. (2000). Intercalation compounds involving inorganic layered structures. An. Acad. Bras. Cienc..

[69] Jacobson A.J., Johnson J.W., Lewandowski J. (1987). Intercalation of the layered solid acid HCa_2_Nb_3_O_10_ by organic amines. Mater. Res. Bull..

[70] Minich I.A., Silyukov O.I., Kurnosenko S.A., Gak V.V., Kalganov V.D., Kolonitskiy P.D., Zvereva I.A. (2021). Physical–Chemical Exfoliation of *n*-Alkylamine Derivatives of Layered Perovskite-like Oxide H_2_K_0.5_Bi_2.5_Ti_4_O_13_ into Nanosheets. Nanomaterials.

[71] Tahara S., Sugahara Y. (2003). Interlayer Surface Modification of the Protonated Triple-Layered Perovskite HCa_2_Nb_3_O_10_·xH_2_O with *n*-Alcohols. Langmuir.

[72] Tahara S., Ichikawa T., Kajiwara G., Sugahara Y. (2007). Reactivity of the Ruddlesden−Popper Phase H_2_La_2_Ti_3_O_10_ with Organic Compounds: Intercalation and Grafting Reactions. Chem. Mater..

[73] Kurnosenko S.A., Voytovich V.V., Silyukov O.I., Minich I.A., Malygina E.N., Zvereva I.A. (2021). Inorganic-organic derivatives of layered perovskite-like titanates HLnTiO_4_ (Ln = La, Nd) with *n*-amines and *n*-alcohols: Synthesis, thermal, vacuum and hydrolytic stability. Ceram. Int..

[74] Wang C., Tang K., Wang D., Liu Z., Wang L., Zhu Y., Qian Y. (2012). A new carbon intercalated compound of Dion–Jacobson phase HLaNb_2_O_7_. J. Mater. Chem..

[75] Aznar A.J., Sanz J., Ruiz-Hitzky E. (1992). Mechanism of the grafting of organosilanes on mineral surfaces. IV. Phenylderivatives of sepiolite and poly(organosiloxanes). Colloid Polym. Sci..

[76] Shimada A., Yoneyama Y., Tahara S., Mutin P.H., Sugahara Y. (2009). Interlayer surface modification of the protonated ion-exchangeable layered perovskite HLaNb_2_O_7_xH_2_O with organophosphonic acids. Chem. Mater..

[77] Machida M., Mitsuyama T., Ikeue K., Matsushima S., Arai M. (2005). Photocatalytic property and electronic structure of triple-layered perovskite tantalates, MCa_2_Ta_3_O_10_ (M = Cs, Na, H, and C_6_H_13_NH_3_). J. Phys. Chem. B.

[78] Rodionov I.A., Maksimova E.A., Pozhidaev A.Y., Kurnosenko S.A., Silyukov O.I., Zvereva I.A. (2019). Layered Titanate H_2_Nd_2_Ti_3_O_10_ Intercalated With n-Butylamine: A New Highly Efficient Hybrid Photocatalyst for Hydrogen Production From Aqueous Solutions of Alcohols. Front. Chem..

[79] Voytovich V.V., Kurnosenko S.A., Silyukov O.I., Rodionov I.A., Minich I.A., Zvereva I.A. (2020). Study of *n*-alkylamine Intercalated Layered Perovskite-Like Niobates HCa_2_Nb_3_O_10_ as Photocatalysts for Hydrogen Production From an Aqueous Solution of Methanol. Front. Chem..

[80] Voytovich V.V., Kurnosenko S.A., Silyukov O.I., Rodionov I.A., Bugrov A.N., Minich I.A., Malygina E.N., Zvereva I.A. (2021). Synthesis of *n*-Alkoxy Derivatives of Layered Perovskite-Like Niobate HCa_2_Nb_3_O_10_ and Study of Their Photocatalytic Activity for Hydrogen Production from an Aqueous Solution of Methanol. Catalysts.

[81] Kurnosenko S.A., Voytovich V.V., Silyukov O.I., Rodionov I.A., Kirichenko S.O., Minich I.A., Malygina E.N., Khramova A.D., Zvereva I.A. (2021). Photocatalytic Activity of *n*-Alkylamine and *n*-Alkoxy Derivatives of Layered Perovskite-like Titanates H_2_Ln_2_Ti_3_O_10_ (Ln = La, Nd) in the Reaction of Hydrogen Production from an Aqueous Solution of Methanol. Catalysts.

[82] Asahi R., Morikawa T., Ohwaki T., Aoki K., Taga Y. (2001). Visible-light photocatalysis in nitrogen-doped titanium oxides. Science.

[83] Al-Azri Z.H.N., Chen W.T., Chan A., Jovic V., Ina T., Idriss H., Waterhouse G.I.N. (2015). The roles of metal co-catalysts and reaction media in photocatalytic hydrogen production: Performance evaluation of M/TiO_2_ photocatalysts (M = Pd, Pt, Au) in different alcohol-water mixtures. J. Catal..

[84] Zhao G., Busser G.W., Froese C., Hu B., Bonke S.A., Schnegg A., Ai Y., Wei D., Wang X., Peng B. (2019). Anaerobic Alcohol Conversion to Carbonyl Compounds over Nanoscaled Rh-Doped SrTiO_3_ under Visible Light. J. Phys. Chem. Lett..

[85] Shen Z., Hu Y., Li B., Zou Y., Li S., Wilma Busser G., Wang X., Zhao G., Muhler M. (2021). State-of-the-art progress in the selective photo-oxidation of alcohols. J. Energy Chem..

[86] Li B., Hong J., Ai Y., Hu Y., Shen Z., Li S., Zou Y., Zhang S., Wang X., Zhao G. (2021). Visible-near-infrared-light-driven selective oxidation of alcohols over nanostructured Cu doped SrTiO_3_ in water under mild condition. J. Catal..

[87] Wads I. (1962). Photodegradation of Carbohydrates. Part IV. Direct Photolysis of D-Glucose in Aqueous Solution. Acta Chem. Scand..

