# Supplementary material for: Photocatalytic Hydrogen Production from Aqueous Solutions of Glucose and Xylose over Layered Perovskite-like Oxides HCa2Nb3O10, H2La2Ti3O10 and Their Inorganic-Organic Derivatives
Source: Nanomaterials (Basel). 2022 Aug 7;12(15):2717. doi: 10.3390/nano12152717 (PMC9370262; doi:10.3390/nano12152717)
Supplement: Supplementary file 1 [file nanomaterials-12-02717-s001.zip › nanomaterials-1841222-supplementary.pdf]

Supporting Information S1

Scheme and operation principle of photocatalytic setting

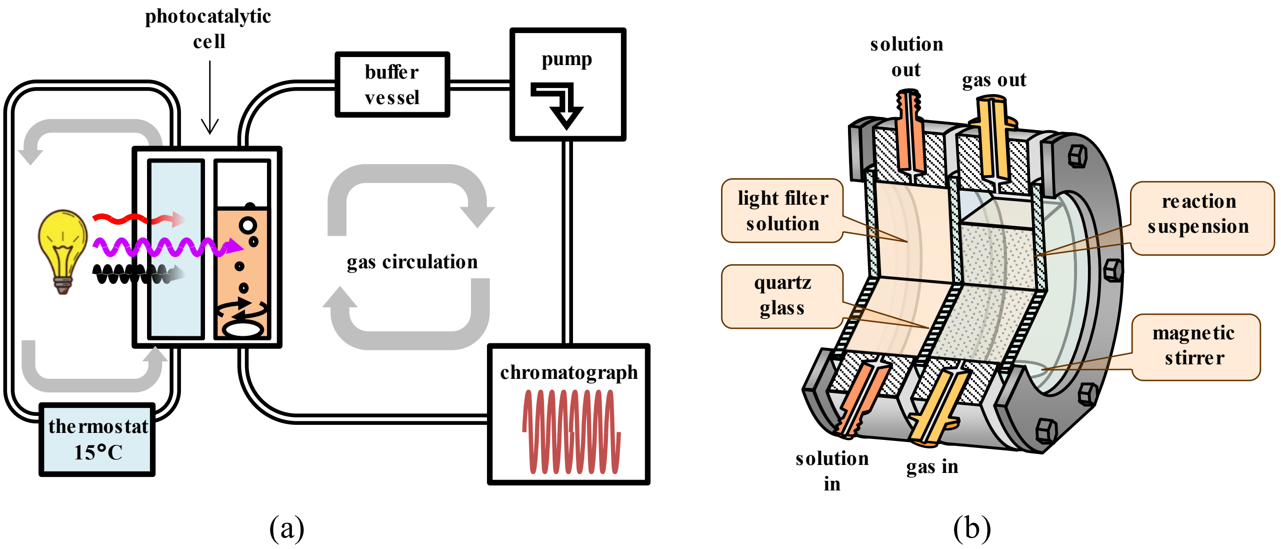

Figure S1. Scheme of photocatalytic setting (a) and reaction cell (b)

Photocatalytic measurements were performed on the laboratory photocatalytic setting, consisting of an illuminator, photocatalytic cell, online gas chromatograph and closed gas circulation system, connecting the last two components. An arc mercury tube lamp DRT-125 (125 W) serves as an ultraviolet irradiation source providing a photon flux of 12.1 mmol/h in the range of the photocatalysts' absorption (spectrum in shown in Supporting Information S2). The self-made photocatalytic cell represents an external irradiation reactor, including two compartments equipped with branch pipes for the supply and removal of solutions and gases. The compartment closest to the lamp is filled with an aqueous solution of NaBr and KCl (6 g/L of each salt) serving as a light filter. The latter allows cutting of hard ultraviolet irradiation ($\lambda < 220$ nm) as well as provides cooling of the cell, which is achieved by its continuous circulation through the thermostat, connected to the cold-water system and maintaining a temperature of 15 °C. The compartment farthest from the lamp is for the reaction suspension, being intensively stirred during the experiment. Gaseous reaction products come from the cell into the gas circulation system. Their detection is conducted according to a specified program at fixed time intervals by a Shimadzu GC-2014 gas chromatograph (Kyoto, Japan), equipped with a 30 m long Rt-Msieve 5A capillary column, thermal conductivity detector and using argon as a carrier gas.

Supporting Information S2

Method for calculation of apparent quantum efficiency

Apparent quantum efficiency φ of photocatalytic hydrogen generation was calculated using the following formula:

$$\varphi = \frac{2\omega}{f} \cdot 100\% \quad (1)$$

where ω is the hydrogen evolution rate (mmol/h) and f is photon flux in the photocatalyst absorption range (mmol/h).

The photon flux was measured previously in accordance with the conventional ferrioxalate actinometry technique. Briefly, 3 g of potassium ferrioxalate $K_3[Fe(C_2O_4)_3]\cdot 3H_2O$ was dissolved in 1 L of 0.05 M sulfuric acid. 50 mL of the photosensitive solution prepared was pumped into the reaction compartment of the photocatalytic cell and irradiated by the DRT-125 lamp through the NaBr + KCl light filter (transmission range $\lambda > 220$ nm) for 20 s. Then 0.5 mL of the irradiated solution was sampled from the cell, mixed with 2 mL of an acetate buffer and 1 mL of 0.1% 1,10-phenanthroline and made up to the 25 mL mark with distilled water in a volumetric flask. Amount of Fe^{2+} in the final solution was determined spectrophotometrically using a previously built calibration plot. The similar experiment was conducted using a KNO_3 light filter and irradiation time 30 s (transmission range $\lambda > 340$ nm). Each series of measurements was repeated 3 times and Fe^{2+} contents were averaged. Assuming the quantum yield of the Fe^{2+} formation from potassium ferrioxalate is 100%, we calculated the photon flux f in the active range (220–340 nm) in accordance with the formula:

$$f = V \cdot \left(\frac{c}{t} - \frac{c'}{t'}\right) \quad (2)$$

where V is a volume of the photosensitive solution in the cell (L), c is concentration of Fe^{2+} (mM) in the photosensitive solution after its irradiation through the NaBr + KCl light filter for the time t (h), c' is concentration of Fe^{2+} (mM) in the photosensitive solution after its irradiation through the KNO_3 light filter for the time t' (h).

Supporting Information S3

Spectrophotometric calibrations for express measurement of reaction suspensions' concentrations

To prepare calibration plots for HCN_3- (a) and HLT_3-based samples (b) for the determination of their concentrations c_1 and c_2 during photocatalytic experiments, a hitch of the protonated form HCN_3 or methylamine derivative $HLT_3 \times MeNH_2$, containing by calculation 0.03 g of the inorganic component (excluding intercalated water), respectively, was added to 60 ml of 1 mol. % aqueous methanol. Then the mixture was sonicated for 10 min in an Elmasonic S10H bath (60 W). Afterwards, the suspension obtained was used to build the spectrophotometric calibration dependence in coordinates optical density (A) – oxide concentration in mg/L (c). For this, a series of spectra with various suspension dilutions was recorded, analytical wavelength λ = 550 nm was selected and linear approximation of the experimental dependence $A_\lambda = A_\lambda\ (c)$ was found using the least-squares method.

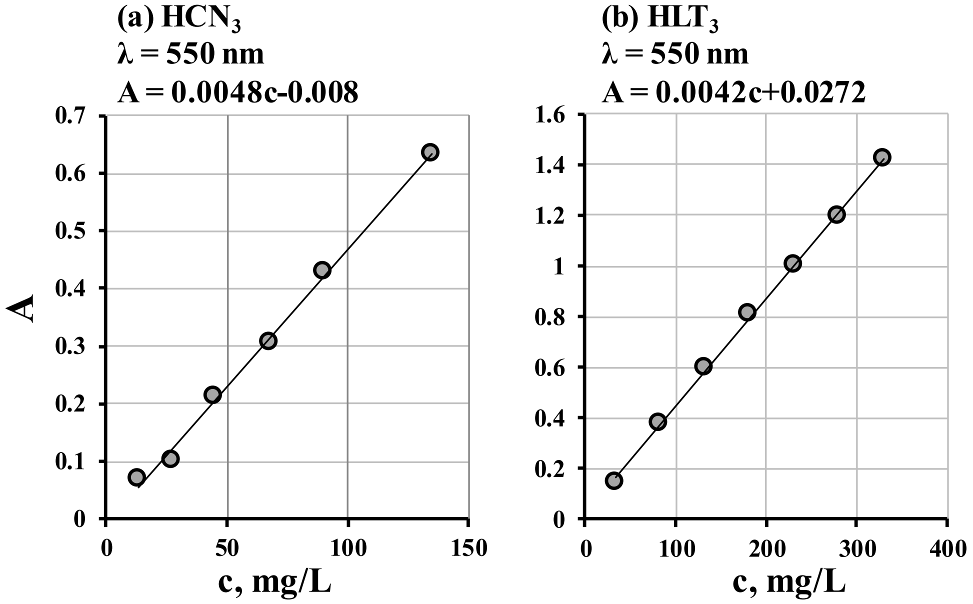

Figure S2. Spectrophotometric calibration plots for the exfoliated forms of HCN_3 (a) and HLT_3 (b)

Table 2

Supporting Information S4

Emission spectrum of the DRT-125 mercury lamp measured through the employed light filter solution compared with the absorption regions of the photocatalysts under study

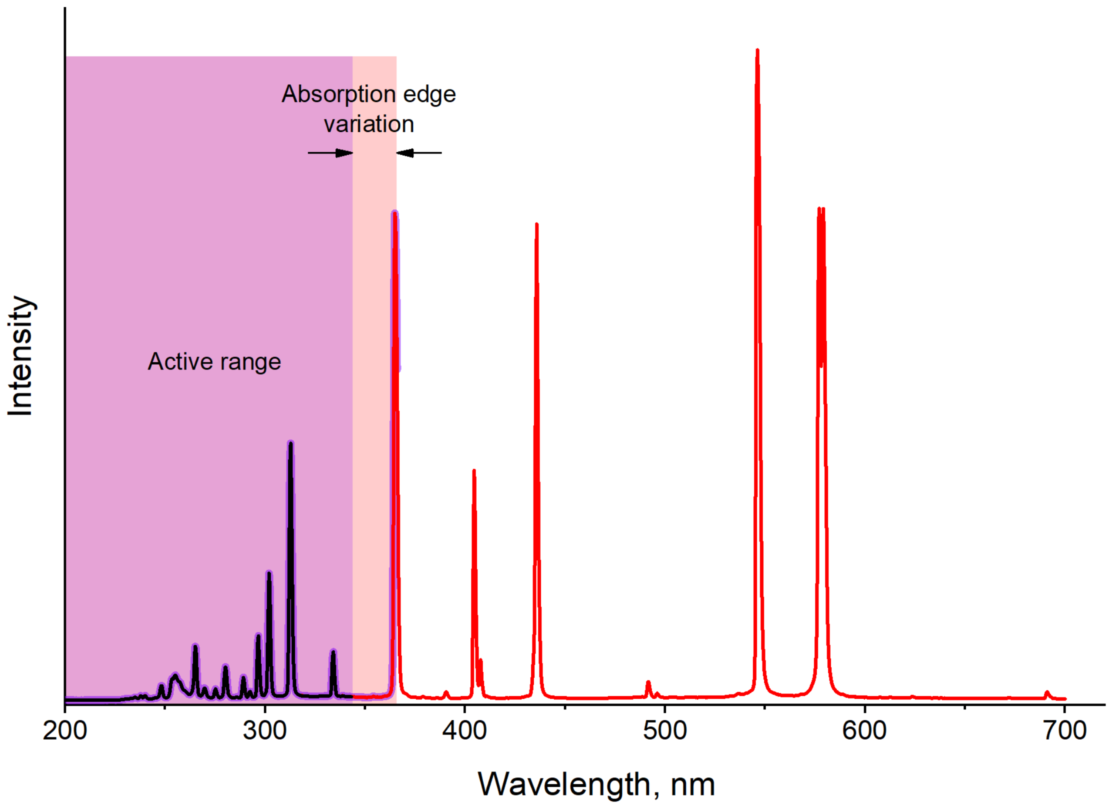

Figure S3. Spectrum of DRT-125 lamp and photocatalysts' absorption range

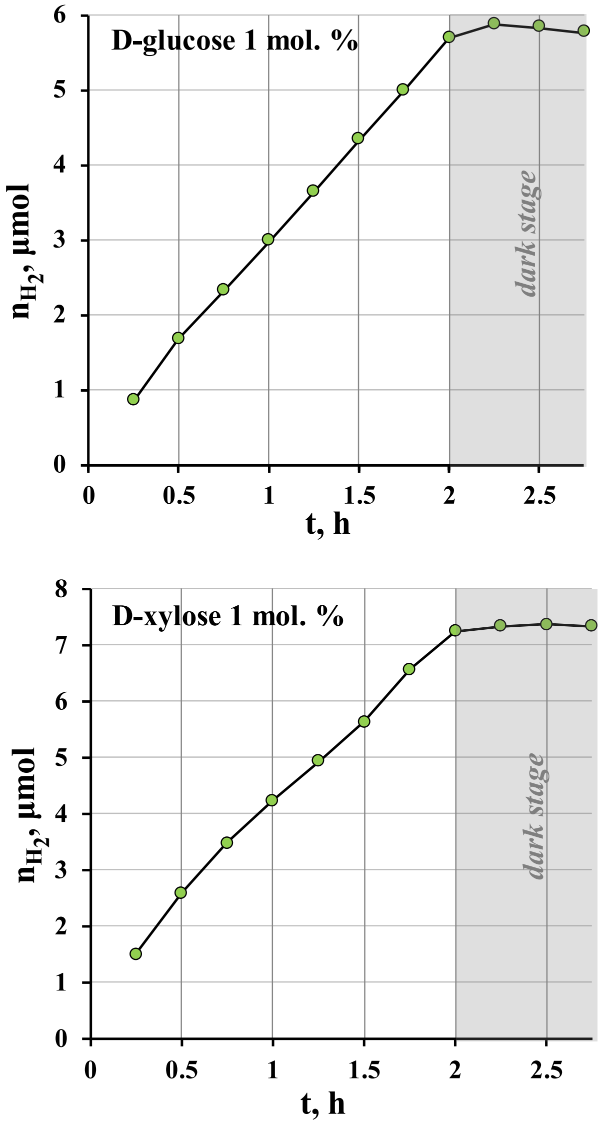

Figure S4. Kinetic curves of photolytic hydrogen evolution from 1 mol. % aqueous carbohydrates under ultraviolet irradiation ($\lambda > 220$ nm)

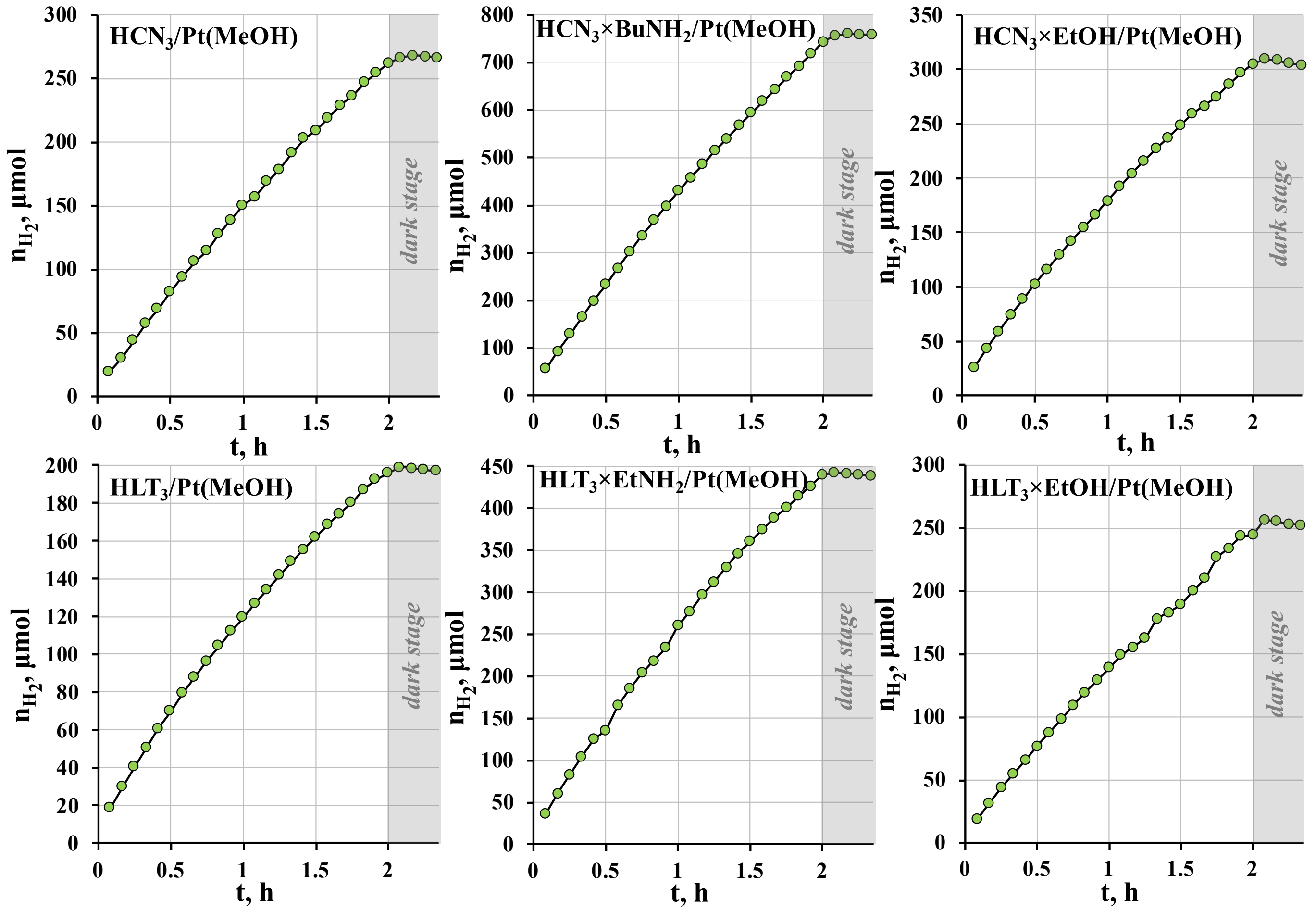

Figure S5. Kinetic curves of photocatalytic hydrogen generation from 1 mol. % aqueous D-glucose over HCN_3- and HLT_3-based samples previously platinized in aqueous.

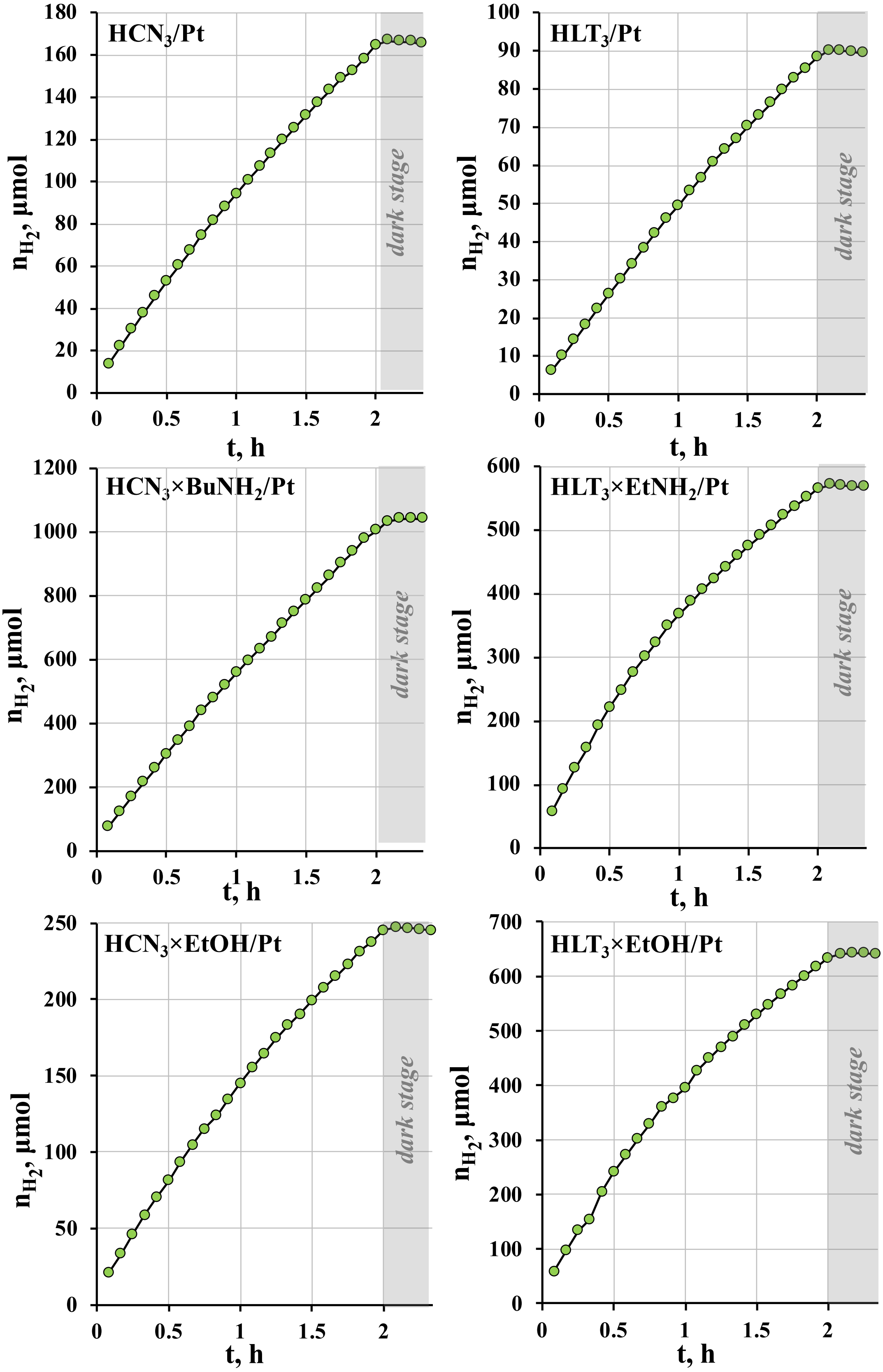

Figure S6. Kinetic curves of photocatalytic hydrogen generation from 1 mol. % aqueous D-xylose over HCN_3- and HLT_3- based samples platinized in situ.

Supporting Information S8

Table S1. Actual volume concentrations of the samples in suspensions and pH of the reaction medium

	Sample	c_1, mg/L	c_2, mg/L	pH_1	pH_2	pH_3
D-glucose	HCN_3	187	86	4.7	4.6	4.5
	HCN_3/Pt	188	149	4.4	4.1	4.0
	HCN_3/Pt(MeOH)	281	201	4.4	4.1	4.1
	$HCN_3 \times BuNH_2$	442	489	7.5	4.9	4.8
	$HCN_3 \times BuNH_2$/Pt	446	426	5.2	3.7	3.6
	$HCN_3 \times BuNH_2$/Pt(MeOH)	500	439	4.4	3.7	3.7
	HCN_3 × EtOH	312	304	4.4	4.3	4.3
	HCN_3 × EtOH/Pt	314	307	4.2	3.9	3.9
	HCN_3 × EtOH/Pt(MeOH)	323	267	4.3	4.0	4.0
	HLT_3	283	111	5.4	5.1	5.0
	HLT_3/Pt	234	140	4.6	4.3	4.2
	HLT_3/Pt(MeOH)	304	148	5.3	4.4	4.3
	$HLT_3 \times EtNH_2$	407	281	4.6	4.4	4.3
	$HLT_3 \times EtNH_2$/Pt	500	219	4.2	3.9	3.9
	$HLT_3 \times EtNH_2$/Pt(MeOH)	257	135	4.9	4.2	4.1
	HLT_3 × EtOH	285	80	5.0	4.5	4.3
	HLT_3 × EtOH/Pt	219	137	4.4	4.0	3.9
	HLT_3 × EtOH/Pt(MeOH)	284	78	5.3	4.4	4.3
D-xylose	HCN_3/Pt	155	151	4.4	4.1	4.0
	$HCN_3 \times BuNH_2$/Pt	437	413	4.6	3.5	3.5
	HCN_3 × EtOH/Pt	310	318	4.3	4.0	3.9
	HLT_3/Pt	252	186	4.6	4.3	4.2
	$HLT_3 \times EtNH_2$/Pt	364	127	4.2	3.9	3.8
	HLT_3 × EtOH/Pt	96	102	4.4	3.9	3.9

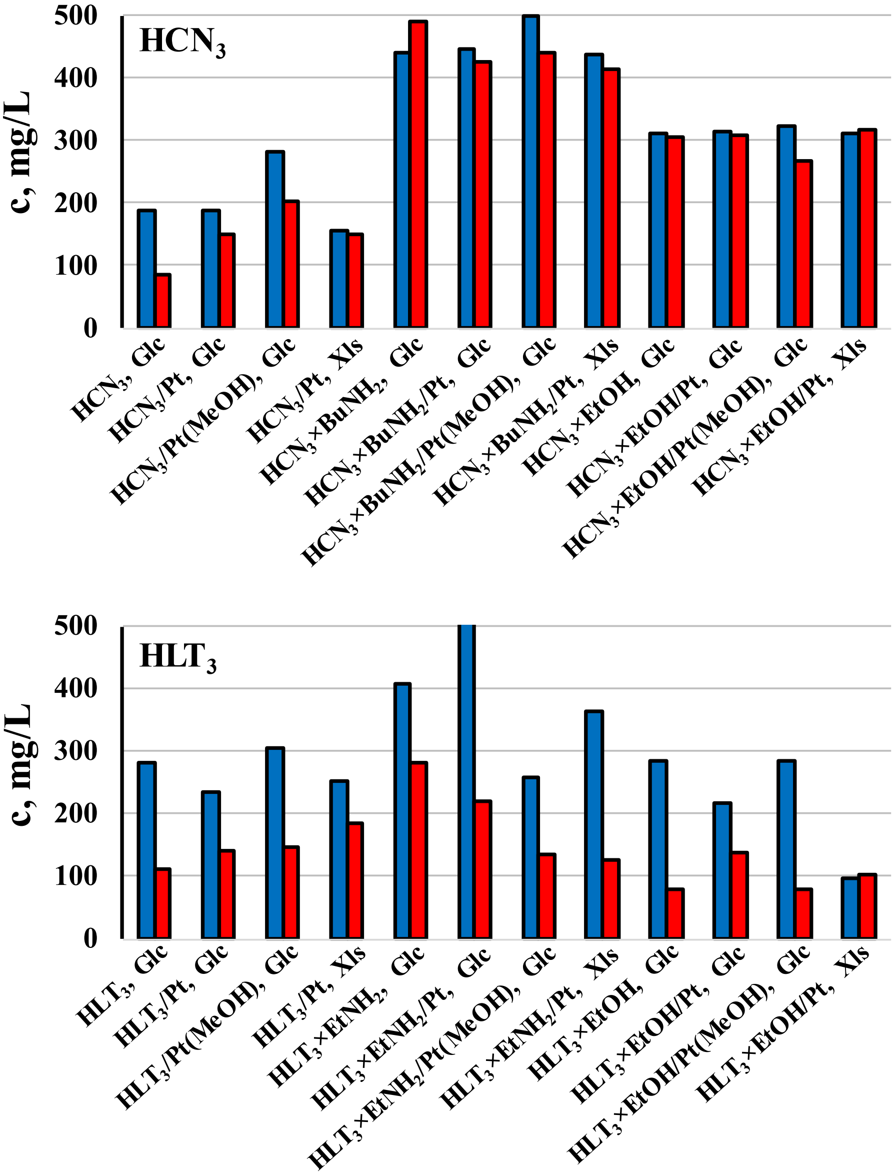

Figure S7. Comparison of actual concentrations of the samples in the reaction suspensions in the beginning (c_1, blue) and in the ending (c_2, red) of the photocatalytic measurement.
